# Intratumoral heterogeneity of surrogate molecular subtypes in urothelial carcinoma in situ of the urinary bladder: implications for prognostic stratification of high-risk non-muscle-invasive bladder cancer

**DOI:** 10.1007/s00428-021-03054-0

**Published:** 2021-03-01

**Authors:** Stefan Garczyk, Felix Bischoff, Ursula Schneider, Reinhard Golz, Friedrich-Carl von Rundstedt, Ruth Knüchel, Stephan Degener

**Affiliations:** 1 Institute of Pathology, University Hospital RWTH Aachen, Pauwelsstr. 30, 52074 Aachen, Germany; 2 Institute of Pathology, Helios University Hospital Wuppertal, Wuppertal, Germany; 3 Department of Urology, Helios University Hospital Wuppertal, Wuppertal, Germany

**Keywords:** NMIBC, Bladder cancer, CIS, Carcinoma in situ, Molecular subtypes, Intratumoral heterogeneity

## Abstract

Reliable factors predicting the disease course of non-muscle-invasive bladder cancer (NMIBC) with carcinoma in situ (CIS) are unavailable. Molecular subtypes have potential for prognostic stratification of muscle-invasive bladder cancer, while their value for CIS patients is unknown. Here, the prognostic impact of both clinico-pathological parameters, including CIS focality, and immunohistochemistry-based surrogate subtypes was analyzed in a cohort of high-risk NMIBC patients with CIS. In 128 high-risk NMIBC patients with CIS, luminal (KRT20, GATA3, ERBB2) and basal (KRT5/6, KRT14) surrogate markers as well as p53 were analyzed in 213–231 biopsies. To study inter-lesional heterogeneity of CIS, marker expression in independent CIS biopsies from different bladder localizations was analyzed. Clinico-pathological parameters and surrogate subtypes were correlated with recurrence-free (RFS), progression-free (PFS), cancer-specific (CSS), and overall survival (OS). Forty-six and 30% of CIS patients exhibited a luminal-like (KRT20-positive, KRT5/6-negative) and a null phenotype (KRT20-negative, KRT5/6-negative), respectively. A basal-like subtype (KRT20-negative, KRT5/6-positive) was not observed. A significant degree of inter-lesional CIS heterogeneity was noted, reflected by 23% of patients showing a mixed subtype. Neither CIS surrogate subtype nor CIS focality was associated with patient outcome. Patient age and smoking status were the only potentially independent prognostic factors predicting RFS, PFS, OS, and PFS, respectively. In conclusion, further clarification of heterogeneity of surrogate subtypes in HR NMIBC and their prognostic value is of importance with regard to potential implementation of molecular subtyping into clinical routine. The potential prognostic usefulness of patient age and smoking status for high-risk NMIBC patients with CIS needs further validation.

**Supplementary Information:**

The online version contains supplementary material available at 10.1007/s00428-021-03054-0.

## Introduction

Bladder cancer (BC) is the most common malignancy of the urinary tract: an estimated number of 549,000 new cases and 200,000 deaths were registered in 2018 worldwide [1]. Muscle-invasive bladder cancer (MIBC) has an unfavorable prognosis (5-year survival <50%) and mainly develops from carcinoma in situ (CIS), a flat-growing, high-grade (HG) lesion characterized by frequent *TP53* alterations [2].

Based on the current treatment guidelines by the European Association of Urology (EAU) and the American Urological Association (AUA) [3, 4], first-line intravesical bacillus Calmette-Guérin (BCG)-based immunotherapy following transurethral resection (TUR) of the tumor is recommended for treatment of high-risk (HR) non-MIBC (NMIBC), including patients exhibiting CIS lesions. However, BCG therapy fails in a substantial number of patients [5] caused by cessation of the therapy due to resistance or toxicity [6, 7]. Radical cystectomy (RC) is recommended as second-line treatment in case of BCG failure [3, 4]. Although associated with an excellent tumor-specific survival [8], RC is a morbid surgery significantly impacting quality of life and thus not all patients are eligible or refuse RC [7]. Moreover, the RC-related rate of overtreatment is assumed to be high [5] due to a lack of reliable prognostic markers [3]. Obviously, there is a need for both, alternative bladder-preserving therapies and reliable prognostic markers allowing the identification of HR NMIBC patients with worse prognosis that might benefit from timely RC and those profiting most from conservative treatment.

Extensive molecular characterization of MIBC has led to the identification of different subtypes with divergent clinical outcomes. In general, a major luminal subgroup associated with favorable and a basal subtype exhibiting worse survival was discovered [9, 10]. Importantly, immunohistochemistry-based subgrouping by surrogate markers might allow feasible implementation of subtype stratification into clinical routine [11]. Regarding non-muscle-invasive urothelial cancer, preliminary data indicate that especially luminal-like urothelial lesions (of the upper tract and the urinary bladder) might be related with worse prognosis compared to those with basal phenotype [12, 13].

With regard to the lack of reliable prognostic stratification markers for HR NMIBC patients presenting with aggressive CIS lesions [3], we sought to analyze the potential prognostic impact of both clinico-pathological parameters including CIS focality and additionally immunohistochemistry-based surrogate subtypes of bladder cancer in a cohort of HR NMIBC patients with CIS. To the best of our knowledge, this is the first study analyzing the potential prognostic value of the recently described molecular subtypes, defined by immunohistochemical surrogate markers, in CIS lesions of HR NMIBC patients.

## Materials and methods

### Patient cohort

We retrospectively analyzed all patients (*n*=2.792) undergoing transurethral bladder resection (TURBT) at the Urology Department of the Helios University Hospital Wuppertal between 2008 and 2014. In 1.424 of these patients (51%), a urothelial carcinoma was detected. Patients with concurrent or a history of prior MIBC were excluded (*n*=285; 20%). In total, 128 patients (11%) were diagnosed with CIS, with or without concurrent Ta and T1 high-risk NMIBC (Table 1). In all patients with Ta and T1 NMIBC, a re-TURBT was performed after 4–6 weeks. The NMIBC follow-up was performed according to the EAU NMIBC guideline [3]. A histological confirmation of a recurrence was mandatory during the follow-up. All clinico-pathological and follow-up data were obtained from the hospital records and two experienced uropathologists (RK and RG) reviewed the histological specimens of all patients to confirm the diagnosis. The median follow-up of the patient cohort was 66 months (range: 3–122 months). The study was conducted at the Helios University Hospital Wuppertal and the University Hospital RWTH Aachen in accordance with the requirements of the institutional review board of the University of Witten/Herdecke (No. 55/2019), the current version of the Declaration of Helsinki and the good clinical practice guidelines.

**Table 1:** Characteristics of HR NMIBC patients with CIS

	Number *n*=128	%
Age (median: 72 years, range: 44–89 years)		
≤ 72	65	51
> 72	63	49
Sex		
Male	107	84
Female	21	16
Smoking status		
Never	70	55
Former	31	24
Current	27	21
Prior UC^a^		
Yes	37	29
No, primary	91	71
Prior recurrence rate^b^		
Primary	91	71
≤ 1 recurrence/yr	23	18
> 1 recurrence/yr	13	10
unknown RR	1	1
Prior intravesical therapy		
BCG	12	9
Mitomycin C	1	1
none	115	90
BCG therapy^c^		
No	38	30
Yes	90	70
CIS focality		
Unifocal	53	41
Multifocal	75	59
CIS clinical type		
Isolated CIS	24	19
Concurrent CIS	104	81
Concomitant pTa LG		
Unifocal	11	9
Multifocal	1	1
None	116	91
Concomitant pTa HG		
Unifocal	36	28
Multifocal	23	18
None	69	54
Concomitant pT1		
Unifocal	45	35
Multifocal	15	12
None	68	53
HG tumor focality		
Unifocal	31	24
Multifocal	97	76
Recurrence at first follow-up		
No	112	88
Yes	16	13
Recurrence		
No	78	61
Yes	50	39
Progression		
No	106	83
Yes	22	17
Survival		
Dead	55	43
Alive	72	56
Unknown	1	1
Cause of death		
UC	16	29
Other	30	55
Unknown cause of death	9	16
BCG response		
Treatment success (including late relapse^d^)	63	70
BCG-unresponsive (including early relapse^e^)	15	17
BCG-failure	10	11
BCG-intolerant	1	1
Not specified	1	1
Radical cystectomy		
No	90	70
Yes	38	30

a: non-muscle-invasive bladder/upper tract urothelial carcinoma; b: low-grade recurrences are included; c: at least induction therapy; d: high-grade recurrence at ≥ 2 years after receipt of adequate BCG; e: high-grade recurrence at 6 months up to < 2 years. Percentages may not sum up to 100 % due to rounding. *RR*, recurrence rate; *TURB*, transurethral resection bladder; *UC*, urothelial carcinoma

Our retrospective immunohistochemistry (IHC) cohort comprised altogether 266 biopsy samples from 128 patients (75 patients with multifocal, 53 cases with unifocal CIS). Due to differential availability of adequate CIS tissue material on prepared tissue microarrays (TMAs), variable numbers of biopsy samples (ranging from 213–231) were stained and analyzed for each IHC marker (Online Resource 1).

### Immunohistochemistry

Formalin-fixed, paraffin-embedded (FFPE) CIS material was used to create tissue microarrays (TMAs). Positive and negative staining controls were included on all TMAs. CIS-heterogeneity, i.e., inter-lesional heterogeneity of multifocal CIS was considered by analyzing marker expression in independent biopsies taken from different CIS localizations in the same urinary bladder, respectively. In any case with sufficient available biopsy material, we additionally analyzed intra-lesional (intra-localization) heterogeneity by generating two to three TMA cores from the same biopsy. TMA sections (2 μm) were incubated with antigen retrieval solution PT Link (Dako, Agilent, Santa Clara, California) of pH 6 (KRT14, KRT20, GATA3, ERBB2) and pH 9 (KRT5/6 and p53) at 95°C for deparaffinization, rehydration, and epitope retrieval. Slides were subsequently transferred to an automated immunostainer (Dako, Agilent) and covered with EnVision^TM^ Flex Peroxidase Blocking-Reagent (Dako, Agilent) for 5 min. Next, immunostaining was performed using validated antibodies for KRT20, GATA3, ERBB2, KRT5/6, KRT14, and p53 [14, 15]. Subsequently, tissue sections were treated with a secondary reagent (Dako, Agilent) for 15 min, followed by incubation with a horseradish peroxidase-conjugated polymer (Dako, Agilent) for 20 min. Finally, visualization of staining was accomplished using a DAB+ Substrate Chromogen System (Dako, Agilent) and tissue sections were counterstained using Mayer’s hematoxylin.

All immunohistochemical stainings were assessed by an experienced uropathologist (RK). The percentage of positively stained cells was evaluated for the cytoplasmic markers KRT20, KRT5/6, KRT14, and nuclear p53. As described previously [14, 15], KRT20, KRT5/6, and KRT14 immunohistochemistry were evaluated with cutoffs of >50% as positive. This value seems plausible to the experienced uropathologist, since it is sometimes hard to unequivocally exclude reactive changes of basal cells (KRT5/6 and KRT14 positive) and regenerative superficial cells (KRT20 positive), which may well be mixed in with the cells of carcinoma in situ. The fact that CIS can be pagetoid and does not have to include the whole thickness of the urothelium was taken into consideration, when semiquantitative evaluation was carried out. Aberrant p53 expression was assumed if either 100 % of cells exhibited intense nuclear staining or in case of complete absence of nuclear staining [16]. GATA3 expression was assessed using an adapted semi-quantitative immunoreactive scoring system [17], multiplying a score for nuclear staining intensity (from 0 to 3) with a score expressing the percentage of stained cells: 0%=0, <10%=1, 10-50%=2, 50-80%=3, >80%=4. A score ranging from 3-12 was considered “positive” as described recently [14]. The Dako score was used to quantify ERBB2 protein expression, combining staining intensity and the percentage of positive cells: 0-1 (negative), 2 (moderate), and 3 (positive, overexpressed) [18]. In case of analysis of several cores taken from the same biopsy material (intra-lesional heterogeneity), the mean staining results were calculated for cytokeratin and GATA3 expression, whereas the strongest staining result for ERBB2 was selected. P53 staining was considered aberrant if at least one of the cores showed aberrant staining.

### Statistical analysis

Univariate Kaplan-Meier analysis and multivariate Cox proportional hazards regression models were used to identify potential prognostic factors for risk stratification of HR NMIBC patients with CIS. Clinically relevant covariates (based on relevant literature) and variables showing a statistically significant (logrank *p* < 0.1) association with the respective survival endpoint in univariate analysis were included in the multivariate models. A number of at least 10 events per included independent variable was considered in the multivariate models [19]. The level of significance in the multivariate analysis was set to *p* < 0.05. Recurrence-free survival was defined as the time interval from tumor resection at the time of study inclusion to first tumor recurrence, whereas progression-free survival was defined as the time interval from study inclusion to the first increase in stage. Overall and urothelial cancer-specific survival were defined as the time interval from study inclusion to death from any cause and death related to urothelial cancer, respectively. Patients without an event or death were censored at the last date of follow-up. All analyses were conducted by using IBM SPSS Statistics (version 26).

## Results

### Patient characteristics

The main characteristics of the HR NMIBC patients with CIS are summarized in detail in Table 1 and only a few data central to the topic may be pointed out. Twenty-nine percent of patients had a history of prior non-muscle-invasive urothelial carcinoma of the bladder and/or the upper tract. The majority of cases (90%) did not receive prior intravesical therapy and 70% of patients were treated with BCG (at least induction therapy) following the date of inclusion in the retrospective study. Forty-one percent of patients presented with unifocal CIS, whereas 59% exhibited multifocal CIS lesions. The majority of patients exhibited concurrent CIS lesions (81%) and a frequent association was found with papillary high-grade (pTa HG) tumors (46%) and only rarely with papillary low-grade (pTa LG) lesions (10%). By nature of a high-grade lesion, CIS in an identical bladder location as a high-grade papillary tumor cannot be differed from a flat rim of a papillary tumor. Stroma-invasive disease (pT1) was found in 47% of all patients. Isolated CIS lesions were identified in 19% of all patients and of those 29% were primary CIS. BCG treatment success, defined as a disease-free state for at least 2 years after receipt of adequate BCG therapy, was observed in 70% of BCG-treated cases (63/90). Further, 17%, 11% and 1% were categorized as BCG-unresponsive, BCG-failure and BCG-intolerant cases, respectively [7].

### Prognostic impact of clinico-pathologic parameters

The median follow-up for the patient cohort was 66 months (range: 3–122 months). Within this time frame, a portion of 39% (50/128 patients) experienced at least one recurrence, with a median time to first recurrence of 15 months. Seventeen percent of patients (22/128) showed progressive disease, with a median time to progression of 19 months. Forty-three percent of patients (55/128) died within the follow-up period and 29% of deaths were related to urothelial cancer.

In the univariate analysis, the following clinico-pathological parameters were identified to be of potential relevance to predict disease recurrence (patient age), disease progression (smoking status, patient age), urothelial cancer-specific survival (patient age, CIS clinical type, concomitant pT1, BCG therapy) and overall survival (patient age, BCG therapy, recurrence at first follow-up) (Table 2). Due to limited group sizes, the parameter “concomitant pTa LG” was excluded from the univariate analysis.

**Table 2:** Univariate analysis of clinico-pathologic parameters and surrogate molecular CIS subtype

Variable	RFS		PFS		CSS		OS	
	n/event	*p*^a^	n/event	*p*^a^	n/event	*p*^a^	n/event	*p*^a^
Age (years)		*<0.001*		*0.052*		*0.021*		*<0.001*
≤ 72	65/17		65/8		63/5		64/10	
> 72	63/33		63/14		55/11		63/45	
Sex		0.695		0.895		0.978		0.333
Male	107/42		107/18		97/13		106/48	
Female	21/8		21/4		21/3		21/7	
Smoking		0.176		*0.006*		0.524		0.290
Never	70/24		70/10		65/7		70/28	
Former	31/12		31/2		30/5		31/16	
Current	27/14		27/10		23/4		26/11	
Prior UC		0.607		0.184		0.782		0.170
No	91/37		91/13		85/11		90/34	
Yes	37/13		37/9		33/5		37/21	
Prior RR		0.724		0.126		0.503		0.183
Primary	91/37		91/13		85/11		90/34	
≤1	23/9		23/7		20/4		23/14	
>1	13/4		13/2		12/1		13/7	
Prior intravesical therapy		0.527		0.147		0.694		0.564
No	115/44		115/18		106/15		114/48	
Yes	13/6		13/4		12/1		13/7	
BCG therapy		0.301		0.933		*0.077*		*0.048*
No	38/11		38/6		33/7		38/22	
Yes	90/39		90/16		85/9		89/33	
Recurrence at first FU		-		0.623		0.263		*0.022*
No	-		112/19		104/13		111/44	
Yes	-		16/3		14/3		16/11	
CIS focality		0.463		0.301		0.302		0.659
Unifocal	53/18		53/7		51/5		53/22	
Multifocal	75/32		75/15		67/11		74/33	
HG tumor focality		0.476		0.927		0.402		0.245
Unifocal	31/14		31/6		30/3		31/11	
Multifocal	97/36		97/16		88/13		96/44	
CIS clinical type		0.914		0.742		*0.038*		0.174
Isolated	24/10		24/4		22/0		24/8	
Concurrent	104/40		104/18		96/16		103/47	
Concomitant pTa HG		0.792		0.970		0.859		0.825
No	69/28		69/12		63/9		68/31	
Yes	59/22		59/10		53/7		59/24	
Concomitant pT1		0.907		0.521		*0.044*		0.291
No	68/28		68/11		63/5		68/27	
Yes	60/22		60/11		55/11		59/28	
Molecular CIS subtype		0.768		0.779		0.996		0.846
Null	30/11		30/6		28/4		30/12	
Mixed	23/8		23/3		21/3		22/8	
Luminal	46/20		46/9		43/6		46/22	
Molecular CIS subtype		0.488		0.700		0.962		0.564
Non-luminal^b^	53/19		53/9		49/7		52/20	
Luminal	46/20		46/9		43/6		46/22	

*p* values < 0.1 were considered significant and are shown in italics

a: logrank test, b: cases defined to exhibit a “null” and “mixed” subtype respectively; *CSS*, urothelial cancer-specific survival; *FU*, follow-up, *OS*, overall survival, *PFS*, progression-free survival; *RFS*, recurrence-free survival; *RR*, recurrence rate; *UC*, urothelial carcinoma

### Prognostic impact of molecular CIS subtypes

To date, it is unclear if molecular subtypes might have a relevance for prognostic stratification of HR NMIBC as shown for MIBC [10, 20]. Here, we analyzed subtype IHC-surrogate marker expression [11] in the CIS lesion(s) of each HR NMIBC patient with available tissue material, comprising luminal (KRT20, GATA3, ERBB2) and basal (KRT5/6, KRT14) markers as well as p53 as a diagnostic marker. Inter-lesional CIS heterogeneity was considered by analyzing marker expression in independent biopsies taken from different CIS localizations in the same urinary bladder, respectively. In agreement with our previous findings in an independent cohort of isolated CIS cases [14], positivity for luminal markers was observed in the majority of CIS biopsies while predominantly lacking expression of basal cytokeratins (Online Resource 1).

**Fig. 1:**
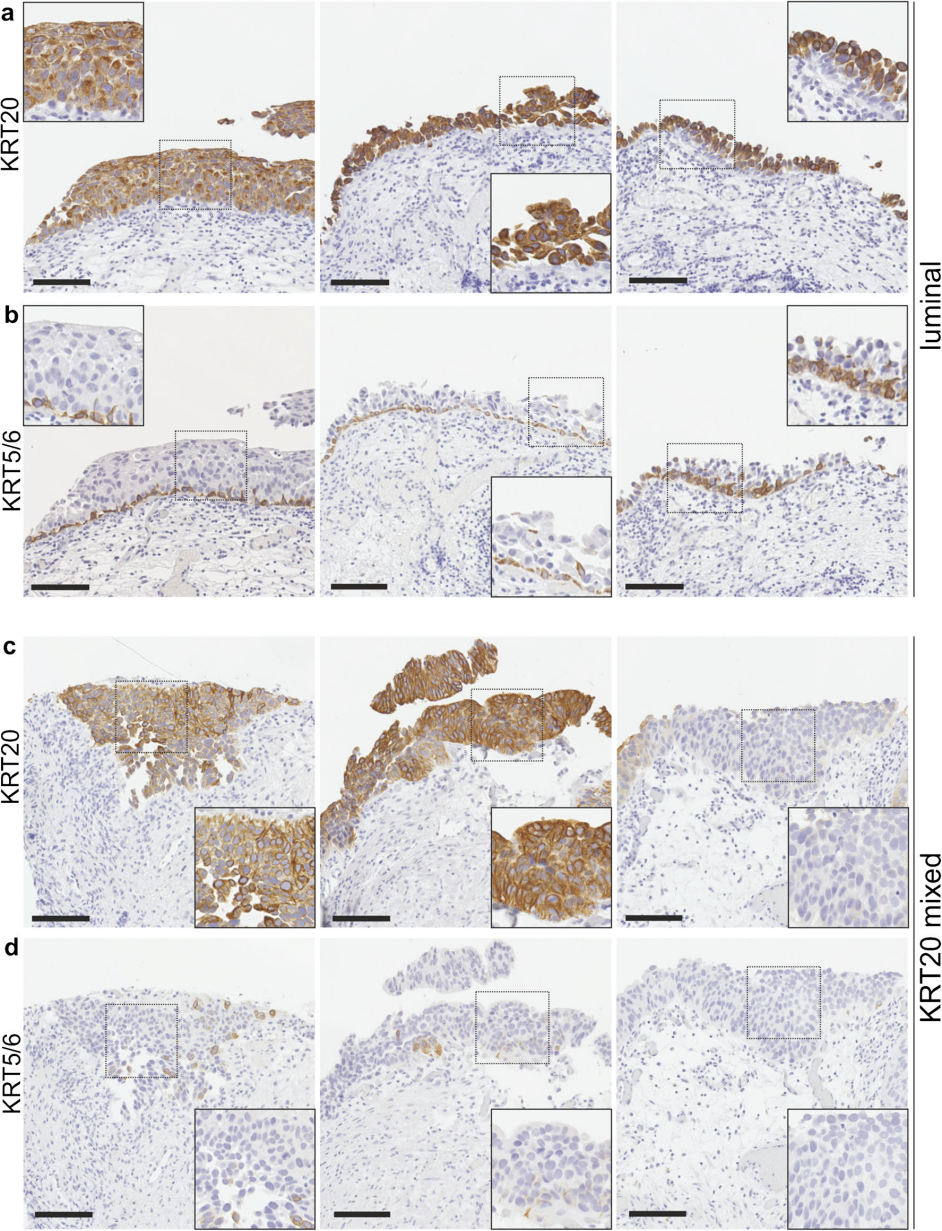
Surrogate CIS subtypes. Based on KRT20 and KRT5/6 expression, high-risk NMIBC patients with CIS were stratified into surrogate subtypes. Exemplary luminal case, characterized by consistent positivity for KRT20 as well as absence of KRT5/6 in the tumor cells in three distinct CIS localizations in the same urinary bladder (**a** and **b**). Exemplary high-risk NMIBC patient with CIS showing a mixed KRT20 phenotype, i.e., KRT20 tumor cell positivity in only two of three distinct CIS localizations in the same urinary bladder but consistent KRT5/6 CIS cell negativity (**c** and **d**). Boxed areas in each micrograph are shown in higher magnification. Scale bars: 100μm

Recently, the potential utility of a two-marker-based approach has been suggested as a prognostic stratification system for MIBC and NMIBC patients [11, 13]. Applying KRT20 and KRT5/6 protein expression as luminal and basal surrogate marker respectively, HR NMIBC patients with CIS were stratified into the three major CIS groups “luminal”, “null” and “mixed,” while the latter comprised four subgroups due to heterogenous inter-lesional marker expression in different CIS localizations of the same patients (Table 3, Fig. 1 and Online Resource 2). Due to unavailability of CIS tissue material on the respective TMAs for KRT20 and KRT5/6 staining, stratification was valid for 99 of 128 patients. Importantly, a “basal-like” subtype (KRT20 negative, KRT5/6 positive) was not observed. The majority (46 %, 46/99 cases) of CIS patients was characterized as “luminal”, whereas 30% (30/99 cases) and 23% (23/99 cases) exhibited a “null” and “mixed” phenotype, respectively. No statistically significant association between surrogate molecular CIS subtype of HR NMIBC patients and survival was noted (Table 2).

**Table 3:** CIS patient stratification into three major groups based on KRT20 and KRT5/6 protein expression

Major CIS group	CIS subgroup	Marker expression	Patients
**-**	**-**	**-**	99 (100 %)
luminal	**-**	KRT20 positive	46 (46 %)
		KRT5/6 negative	
null	**-**	KRT20 negative	30 (30 %)
		KRT5/6 negative	
mixed	**-**	-	23 (23 %)
	1	KRT20 mixed	18
		KRT5/6 negative	
	2	KRT20 mixed	3
		KRT5/6 mixed	
	3	KRT20 negative	1
		KRT5/6 mixed	
	4	KRT20 mixed	1
		KRT5/6 positive

### Multivariate analysis

Depending on the number of events for RFS, PFS, CSS, and OS in the current study, observed significance of factors in the univariate analysis (*p* < 0.1) and recently described prognostic potential of factors for stratification of BCG-treated NMIBC patients associated with CIS [21, 22], the following variables were subsequently included in the multivariate models: RFS (age, sex, prior UC, CIS focality), PFS (smoking status, age), and OS (age, BCG therapy, recurrence at first follow-up, concomitant pT1) (Table 4). Due to a limited number of events, multivariate analysis for CSS was not valid and thus not performed.

**Table 4:** Multivariate analysis

Variable	RFS		PFS		OS	
	HR (95% CI)	*p*	HR (95% CI)	*p*	HR (95% CI)	*p*
Age						
≤ 72	1.00		1.00		1.00	
> 72	3.23 (1.73–6.04)	*<0.001*	3.08 (1.26–7.54)	*0.014*	7.38 (3.63–15.01)	*<0.001*
Sex			-	-	-	-
Female	1.00					
Male	0.78 (0.35–1.77)	0.557				
Prior UC			-	-	-	-
Yes	1.00					
No	1.62 (0.82-3.21)	0.166				
Concomitant pT1	-	-	-	-		
No					1.00	
Yes					1.49 (0.86–2.60)	0.160
CIS focality			-	-	**-**	**-**
Unifocal	1.00					
Multifocal	1.12 0.62–2.01)	0.704				
Smoking status	-	-			-	-
Never			1.00			
Former			0.54 (0.12–2.45)	0.420		
Current			3.91 (1.59–9.62)	*0.003*		
BCG therapy	-	-	-	-		
No					1.00	
Yes					0.62 (0.35–1.09)	0.099
Recurrence at first FU	-	-	-	-		
No					1.00	
Yes					1.50 (0.76–2.97)	0.240

*p* values < 0.05 were considered significant and are shown in italics

*CI*, confidence interval; *FU*, follow-up, *HR*, hazard ratio, *OS*, overall survival, *PFS*, progression-free survival, *RFS*, recurrence-free survival; *UC*, urothelial carcinoma

In the multivariate models, patient age was significantly associated with RFS, PFS, and OS, whereas smoking status was identified as a potential independent predictor of PFS in HR NMIBC patients with CIS.

## Discussion

Currently, no reliable prognostic factors are available to predict the disease course of CIS patients [3]. Thus, there is an unmet need to identify markers allowing stratification of CIS patients into those cases benefiting most from early radical cystectomy and those profiting from conservative treatment.

Existing prognostic scores and risk tables are based on data from studies that were not specifically focused on CIS patients [21, 23, 24]. The European Organisation for Research and Treatment of Cancer (EORTC) scoring system for instance was developed on the basis of survival data from NMIBC patients mostly without CIS, and predominantly treated by chemotherapy [23]. The basis of the CUETO (Club Urologico Espanol de Tratamiento) model are data from NMIBC patients treated by suboptimal BCG therapy and again, patients with CIS only represented a small fraction (10% of patients) [21]. In a more recent study, potential prognostic factors in intermediate and high-risk NMIBC patients treated with 1–3 years maintenance BCG were investigated, however, without inclusion of CIS patients [24]. Indeed, several smaller studies analyzing the prognostic value of different factors specifically in CIS patients, including established clinico-pathological parameters, have been performed. For instance, exhibiting concurrent CIS and T1 lesions compared to primary CIS [25], having an extended CIS [26] and CIS localized in the prostatic urethra [27] have been associated with worse patient outcome. It may be emphasized here that all studies are limited to mere histological diagnosis of CIS as a variable, without any further analysis of the lesional biology.

Molecular subtypes have been described previously in MIBC, resembling those found in breast cancer patients [10, 20]. Importantly, these subtypes seem promising with regard to prognostic stratification of MIBC patients, with basal tumors being potentially associated with a more aggressive behavior in comparison to luminal cancers while putatively responding better to chemotherapy than luminal carcinomas [10, 20].

Far less is known about the impact of these molecular subtypes in NMIBC patients. In contrast to MIBC, first data in NMIBC and non-muscle-invasive urothelial cancer of the upper urinary tract indicate that especially luminal-like tumors are associated with an unfavorable outcome [12, 13, 28–30]. The use of a minimal set of immunohistochemical markers (including KRT20 and KRT5/6) has been demonstrated to be a feasible and reliable approach to reflect intrinsic molecular subtypes (at least) in MIBC samples [11].

In a previous study without clinical follow-up, we have shown that isolated CIS lesions are characterized by the expression of luminal markers including KRT20 and GATA3, whereas lacking the expression of the basal cytokeratins KRT5/6 and KRT14 in the majority of samples [14]. In the current study, we were able to validate our previous observations in a large, independent cohort of HR NMIBC patients with CIS. In addition to our previous analyses, we considered the inter-lesional CIS-heterogeneity of marker expression in CIS patients by analyzing marker expression in different CIS localizations in the same urinary bladders and observed a significant degree of heterogeneity. Using KRT20 and KRT5/6 as luminal and basal surrogate marker respectively, CIS patients were categorized into the three major groups “luminal,” “null,” and “mixed,” while the latter comprised different subgroups due to heterogenous KRT20 and KRT5/6 expression in different CIS localizations. Of note, we did not observe a clear “basal” phenotype in our CIS cohort (KRT20 negative, KRT5/6 positive). In contrast to two recent studies in NMIBC [13, 29], we did not observe a significant association of surrogate CIS subtype with patient outcome. This observation remained stable when using additional cutoffs for KRT20 and KRT5/6 positivity (30% and 80% respectively) (data not shown). These putatively conflicting observations might be explained by significant differences between these studies: First, using RNA data, Breyer et al. focused on surrogate subtypes solely in T1 tumors of NMIBC patients [13]. The second study included stage Ta and T1 NMIBC patients, with a substantial fraction of LG tumors (65%), analyzing surrogate subtypes in Ta and T1 samples [29]. Second, neither of the aforementioned studies considered potential intratumoral heterogeneity (ITH) in Ta and T1 tumors investigated, even though known to be relevant in bladder tumors including heterogeneity with regard to molecular subtypes [31, 32]. While intra-lesional heterogeneity in bladder cancer of different stages and grade, except for CIS, has been studied before [33] it has remained unclear thus far if surrogate subtypes differ when considering distinct tumor localizations present in the same urinary bladder and neither is validated which tumor locus is prognostically informative with regard to molecular subtypes in case of heterogeneity. Data from our previous work suggest that there might be considerable ITH in surrogate molecular subtypes, as a switch from a luminal-like to a more basal-like phenotype was observed during the course of stroma-invasion of CIS lesions [14]. We hypothesize that the surrogate molecular subtype of the highest stage and grade lesion in NMIBC might be prognostically informative and this hypothesis will be analyzed in upcoming studies.

Bladder cancer frequently presents as a multifocal disease, potentially representing different tumor clones of a monoclonal origin [31, 34]. In the current study cohort, 59% and 76% of patients had multifocal CIS lesions and multifocal HG tumors (including papillary HG and pT1 lesions), respectively. It was hypothesized that CIS/HG multifocality is related to worse outcome compared to patients with unifocal CIS/HG tumors. This hypothesis is based on the assumption that a more diverse tumor is able to adapt more efficiently to changing environmental conditions resulting in faster tumor (re-)growth and progression [35]. To our knowledge, the potential prognostic significance of CIS/HG focality in CIS patients is unclear and understudied to date. Surprisingly, neither CIS focality nor HG tumor focality in general were significantly associated with survival in the present cohort. This observation is an accordance with a smaller previous study noting that the extent of CIS is not predictive of recurrence or progression [36]. In contrast, Takenaka and colleagues observed a worse PFS in CIS patients with extended CIS [26].

Additionally, we analyzed the prognostic potential of a larger set of established clinico-pathological factors. Higher patient age was identified as a potential independent prognostic parameter of an unfavorable recurrence-free (RFS), progression-free (PFS) and overall survival (OS) of HR NMIBC patients with CIS. Due to a limited number of events, multivariate analysis for urothelial cancer-specific survival has not been performed. However, a significant association of higher age with worse urothelial cancer-specific survival has been noted in univariate analysis as well. An association of increasing age with worse RFS, PFS, OS, and bladder cancer-specific survival has been observed previously in studies considering NMIBC including patients with CIS [21, 22]. Concerning studies specifically focusing on CIS patients, conflicting observations have been made [26, 37]. Moreover, we noted that current smokers exhibited a significantly worse PFS compared to never smokers and former smokers. Interestingly, no significant difference in PFS was observed between never and former smokers. The value of smoking status as a prognostic stratification marker in non-muscle-invasive bladder is controversial [38, 39]. Focusing specifically on patients with CIS, smoking status has to our knowledge so far not been identified as an independent prognostic factor for PFS and needs further validation [37, 40].

The current study is limited by its retrospective character. Even though the study includes a high number of cases with CIS related to its overall frequency in the bladder, we are aware of case numbers being still fairly small for the observations stated. Even though an obvious degree of inter-lesional CIS heterogeneity with regard to surrogate molecular subtypes was identified in this study, we are aware that the use of tissue microarrays instead of whole tissue slides is a limitation.

In summary, we observed that only patient age and smoking status information were independently associated with outcome of HR NMIBC with CIS. Neither the surrogate molecular subtype of CIS lesions in HR NMIBC patients, nor CIS/HG tumor focality were significantly related to prognosis. Importantly, we identified a considerable degree of inter-lesional CIS heterogeneity with regard to surrogate molecular subtypes and conclude that further clarification of potential heterogeneity in HR NMIBC patients is of high value with regard to potential implementation of molecular subtyping into clinical routine. Moreover, due to the putative transient/heterogenous nature of expression-based molecular subtypes in NMIBC, the prognostic impact of mutational profiles should be considered in upcoming studies.

## Supplementary information

Online Resource 1 Protein expression of luminal and basal markers as well as of p53 in CIS biopsies from HR NMIBC patients of two independent cohorts (PDF 45 kb).

Online Resource 2 Stratification of HR NMIBC patients with CIS into surrogate molecular CIS subtypes based on KRT20 and KRT5/6 protein expression (XLSX 20 kb).

## Data Availability

All data underlying the reported findings are included within the manuscript and its supplements. Raw datasets generated during the current study are available from the corresponding author on reasonable request.
