# Supplementary material for: Intratumoral heterogeneity of surrogate molecular subtypes in urothelial carcinoma in situ of the urinary bladder: implications for prognostic stratification of high-risk non-muscle-invasive bladder cancer
Source: Virchows Arch. 2021 Mar 1;479(2):325–35. doi: 10.1007/s00428-021-03054-0 (PMC8364543; doi:10.1007/s00428-021-03054-0)
Supplement: Supplementary file 1 — Protein expression of luminal and basal markers as well as of p53 in CIS biopsies from HR NMIBC patients of two independent cohorts (PDF 45 kb). [file 428_2021_3054_MOESM1_ESM.pdf]

Virchows Archiv

Intratumoral heterogeneity of surrogate molecular subtypes in urothelial carcinoma in situ of the urinary bladder: implications for prognostic stratification of high-risk non-muscle-invasive bladder cancer

Stefan Garczyk, Felix Bischoff, Ursula Schneider, Reinhard Golz, Friedrich-Carl von Rundstedt, Ruth Knüchel and Stephan Degener

Corresponding author:

Stefan Garczyk, PhD, Institute of Pathology, University Hospital RWTH Aachen, Pauwelsstr. 30, 52074 Aachen, Germany, Email: sgarczyk@ukaachen.de

Online Resource 1 Protein expression of luminal and basal markers as well as of p53 in CIS biopsies from HR NMIBC patients of two independent cohorts [14]

	Current CIS cohort		median	CIS Aachen/Munich cohort n= 156 (100%) samples		median
KRT20						
positive	129	(57%)	positive	132	(85%)	positive
negative	98	(43%)		24	(15%)	
ERBB2						
0-1	31	(14%)	3	55	(35%)	2
2	42	(19%)		51	(33%)	
3	150	(67%)		50	(32%)	
GATA3						
0-2	1	(0.4%)	12	5	(3%)	12
3-12	225	(99.6%)		151	(97%)	
KRT5/6						
positive	7	(3%)	negative	3	(2%)	negative
negative	206	(97%)		153	(98%)	
KRT14						
positive	0	(0%)	negative	2	(1%)	negative
negative	231	(100%)		154	(99%)	
p53						
aberrant	150	(68%)	-	99	(63%)	-
non-aberrant	72	(32%)		57	(37%)	

For KRT20, KRT5/6, and KRT14, percentage of positive cells was determined and biopsy samples were considered positive if > 50% of cells expressed the marker. GATA3 expression was scored using the Remmele score, whereas ERBB2 expression was scored with the Dako score. Note: numbers of analyzed samples for each single marker differ due to availability of sample material.
